# Exploring the Effects of Changes in Dietary Protein Content on Naturally Aging Mice Based on Comprehensive Quantitative Scoring and Metabolomic Analysis

**DOI:** 10.3390/nu17091542

**Published:** 2025-04-30

**Authors:** Xiaohua Zheng, Fan Zhou, Qinren Zhang, Wenxuan Zheng, Fengcui Shi, Ruiding Li, Jingwen Lv, Quanyang Li

**Affiliations:** 1 College of Light Industry and Food Engineering, Guangxi University, Nanning 530004, China; zxhua@st.gxu.edu.cn (X.Z.);; 2 Guangxi Key Laboratory of Longevity Science and Technology, Nanning 530200, China

**Keywords:** dietary protein, metabolomics, aging, mice, inbred C57BL, sex factor, neuroprotection, memory

## Abstract

Background: During aging, protein nutrition has a bidirectional role in regulating healthy lifespan by modulating body metabolism and neurological function. However, the current “low-high” hypothesis on the dynamics of protein requirements is mainly based on male animal models, and its applicability to female physiology (e.g., estrogen fluctuations) is unclear. The present study aims to fill the gap in the study of protein demand dynamics in female naturally aging mice and to investigate the effects of different protein levels on the health status of female C57BL/6J mice at different stages of aging. Methods: In this study, four dietary interventions (high protein, HP; low protein, LP; model test, MT; and control, C) were evaluated by constructing a C57BL/6J female mouse model at three ages, 9 M (9 months), 16 M (16 months), and 20 M (20 months), which are approximately equivalent to 34, 65, and 78 years of age in humans, respectively, to determine the effects on naturally aging mice. The effects of the interventions were quantitatively described by behavioral, neuropathological, oxidative, and inflammatory indices and NMR metabolomics using Principal Component Analysis to construct a comprehensive quantitative scoring method. Results: The comprehensive quantitative scores Fsum was highest in the HP group, lowest in the LP group, and in between in the MT group. The HP intervention showed the most significant improvement in the aged group (20 M) mice, with a 35.2% reduction in avoidance latency (*p* < 0.01) and a 32.9% increase in pyramidal cell density in the hippocampal CA1 region (*p* < 0.05), while the LP intervention led to a cognitive decline in the mice, with an avoidance latency that was prolonged by 15.2% (*p* < 0.05). Metabolomics analysis revealed that mouse samples of all ages showed age-dependent metabolic re-adaptation: the 9 M group may reflect gut microbial metabolism rather than direct host TCA cycle activity, suggesting an indirect association with energy metabolism; an enhanced degradation of branched-chain amino acids (BCAAs) was seen in the middle-aged group (16 M); and amino acid biosynthesis was predominant in the old group (20 M). Conclusions: Female mice have sustained neuromotor benefits to high-protein diets at different aging stages, and the dynamics of their protein requirements differ significantly from those of males. The study reveals the critical role of gender factors in protein nutritional strategies and provides an experimental basis for precise protein supplementation in older women.

## 1. Introduction

Aging is a highly complex biological phenomenon involving the functional degradation of multiple systems [1], among which changes in nutrient metabolism are particularly critical [2]. As an essential nutrient for maintaining life activities, dietary protein is important in aging regulation. However, there are seemingly contradictory opinions in academia about the best strategy for protein intake. On the one hand, it is believed that low protein intake can prolong the lifespan of model organisms [3] by inhibiting the mTORC1 signaling pathway [4], activating autophagy [5], and reducing oxidative stress [6], as shown by Wallerer et al. where a long-term low-protein diet significantly extended the life span and improved the metabolic health of test mice [7]. On the other hand, according to the results of epidemiological studies, it is believed that low protein intake in the elderly population is significantly associated with the risk of muscle decay syndrome and cognitive decline [8,9]. For example, a cohort study in the elderly population found that the rate of muscle mass loss was accelerated in the low-protein-intake group and the risk of developing sarcopenia was nearly doubled [10]. In addition, it has been suggested that high protein intake, while improving muscle mass and nerve function, may increase the burden on the kidneys [11], especially in older individuals [12].

In recent years, researchers have found that the nutritional effect of protein is not only dependent on time (age stage) but also related to the spatial dimension (metabolic requirements of different organs) [13]. Young individuals have a strong ability to activate autophagy through a low-protein diet to optimize metabolic efficiency. By contrast, elderly individuals require higher protein intake to maintain tissue function due to the decline in anabolic ability and reduced regulatory ability [11]. In addition, different organs also have different protein requirements [14]: brain tissue strongly relies on amino acid metabolism to maintain synaptic plasticity [15]. By contrast, excessive protein intake may produce toxic metabolites through intestinal flora fermentation, posing a threat to the cardiovascular system [16].

Combining these results, we believe that human protein requirements may be a dynamic change closely related to individual age, sex, and physiological status. By analyzing the experimental results of male SD rats, our research group proposed the “dynamic hypothesis of protein demand”, where the protein demand of the body presents a “low–high” transition after the age of 50, and the age of 65 is the key transition node [17]. This hypothesis was proposed based on experiments in male SD rat models, but it is unclear whether the same pattern exists in female models. Previous studies mainly focused on male animal samples [17,18] and rarely involved female samples. During the aging process, the metabolic adaptability of female individuals is significantly different from that of males due to dynamic changes in estrogen levels [19]. Due to the sudden decline in estrogen levels in postmenopausal women, metabolic adaptability is significantly weakened [20], mainly manifested as decreased muscle mass and accelerated cognitive function degradation [21]. Therefore, it is necessary to explore the relationship between female protein nutrition level and health status.

Based on previous explorations, this study evaluated the effect of the response of female mice to the dietary intervention of gradient protein at different ages, aiming to further improve protein nutrition mechanism, as well as answer the question “Are there sex-specific protein requirement dynamics in female naturally aging mice?”. Referring to the results of Lehallier et al. [22] on plasma proteomics, which found that there are three key turning points in the human aging trajectory at around 34, 65, and 78 years old, we constructed a 3-month age segment, 9 M (9 months), 16 M (16 months), and 20 M (20 months) [23], corresponding to a human female C57BL/6J mouse model at about 34/65/78 years old [24]. Concerning the protein nutrition level [17] in the previous macrobiotic diet model [25,26], the intervention strategy of 30% lower or higher than the basic level was adopted. After a certain period, samples were detected and collected.

Although the “protein requirement dynamics” hypothesis has been preliminarily validated in male mice, no systematic study has been conducted to assess the changes in protein nutritional requirements of female mice during the aging stage because female mice differ significantly from male mice in terms of fluctuations in estrogen levels and metabolic adaptations in the postmenopausal period. The aim of this study was conducted in naturally aging female C57BL/6J mice at 9 M, 16 M, and 20 M (corresponding to the thresholds of 34, 65, and 78 years of age in humans, respectively), and a graded protein intervention (low, high, and dynamic transitions) combined with behavioral, metabolomic, and histopathological indices was used to systematically investigate the female response to protein levels at different aging stages characteristics of the responses of females to protein levels at different aging stages and their underlying mechanisms and to provide an experimental basis for precise nutritional interventions in aged female mice.

## 2. Materials and Methods

### 2.1. Animal Samples and Experimental Design

**Figure 1:**
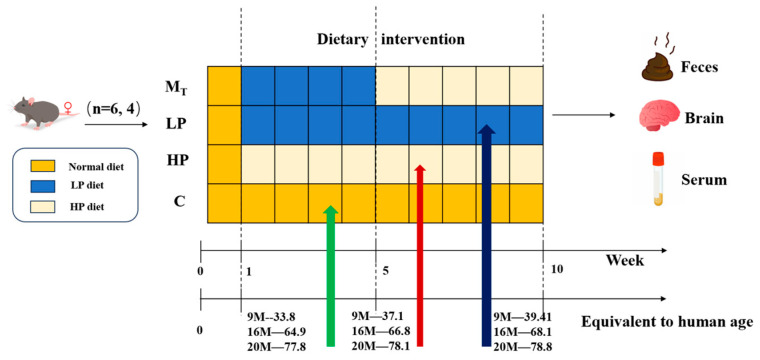
Schematic diagram of animal grouping and intervention experimental design and sample collection.

Test Animal Ethical Review Approval No.: 2023-GXU-249. Details are shown in Figure 1. Twenty-four 9 M, 16 M, and 20 M female C57BL mice (corresponding to humans of 34, 65, and 77.8 years of age) [23] were purchased from Beijing Specific Bio-Technology Co., Ltd. (Test Animal Production License No.: SCXK (Beijing) 2019-0010, Beijing, China). The mice were kept in a room with constant temperature (24 ± 2 °C), maintained at a relative humidity of 60 ± 10%, and controlled with a 12/12 h alternating circadian light/dark cycle, and they were supplied with protein nutrition, food, and water [17] in the same way as in the pre-longevity dietary model [25,26] during the adaptation period; after one week of adaptive feeding, all the mice were randomly divided into four groups (six mice in each group). Under the premise of the same daily dietary calorie intake, they were fed in 4 ways, i.e., the normal control group (C) (basic feed with a protein content of 19.61/100 g feed), the low-protein dietary pattern group (LP) (low-protein feed with protein content decreasing by 30% compared with basic feed, protein content of 13.73/100 g), the high-protein dietary pattern group (HP) (high-protein feed with protein content increasing by 30% compared with basic feed, protein content of 25.49/100 g), and group (M_T_) (switching from an LP to HP diet at week 5, referred to as the model test group).

The mice feed was purchased from Beijing Ke’ao Co-operative Feed Co., Ltd. (Beijing, China). To ensure that the daily dietary calorie intake of mice in each group was the same, the LP and HP feeds were adjusted according to the ratio of the total energy to obtain the total energy consistent with that of the standard feeds, made from the standard chow Ain93 as the base, with corn starch, maltodextrin, whey protein, sucrose, cellulose, lard, mixed vitamin V10037, mixed mineral S10022G, and hydrocholine bitartrate as the configuration ingredients. After 10 weeks of the animal experiment, fecal samples were collected from the mice. After testing the behavioral indices, the experimental mice were dissected, and samples such as serum and visceral tissues were collected.

### 2.2. Absenteeism Experiment of Mouse Samples

The open-field experiment was conducted concerning the literature [17] and slightly modified. The test device mainly consisted of a blue medical ABS plastic box with specifications of 100 cm × 100 cm × 40 cm, Super Maze animal behavior analysis software, and an infrared camera to monitor the behavioral trajectory of the mice. The open-field experiment was carried out after 10 weeks of dietary intervention in mice. The mice were brought into the experimental field 3 h in advance to adapt to the new environment, keeping low light illumination and a quiet atmosphere. Each mouse was put into the four corners of the open field from the four corners of the field. The average moving speed of the mice, the total number of times entering the central area. The number of times the mice’s erection was recorded within 5 min in the open field, and the data were processed and analyzed through the Super Maze animal behavioral analysis software. The data were analyzed by Super Maze animal behavior analysis software for statistics and trajectory processing. The plastic box was wiped with 75% alcohol at the end of each trial to remove the odor of the previous mouse.

### 2.3. Brain Sections of Mouse Samples

Brain tissues were preserved in 4% paraformaldehyde (Hebei Bohai Biological Engineering Development Co., Ltd., Shijiazhuang, China) immediately after the mice were killed. The samples were sealed by dehydration, paraffin embedding, sectioning, patching, hematoxylin–eosin (H&E) staining, and neutral resin. Then, the hippocampus was observed under the microscope.

### 2.4. Quantitative Analysis of MDA, T-AOC in Brain Tissue and Serum IL-10, TNF-α of Mouse Samples

Brain tissues were immediately snap-frozen in liquid nitrogen and stored at −80 °C. Brain tissues were removed and stored in liquid nitrogen on the day of measurement. On the day of measurement, brain tissues were removed, accurately weighed, and homogenized in an ice-water bath by adding 4 times the volume of saline to prepare a homogenate of brain tissues. The MDA and T-AOC contents of brain tissues were measured using MDA and T-AOC kits (Nanjing Jianchen Bioengineering Institute, Nanjing, Jiangsu, China), respectively. IL-10 and TNF-α inflammatory factors in mouse serum were detected using the mouse enzyme immunoassay kit IL-10 and TNF-α kit (Shanghai Jianglai Biotechnology Co., Ltd., Shanghai, China).

### 2.5. Non-Targeted Metabolomics Analysis of Mouse Fecal Samples Based on ^1^H-NMR

(1) Preparation of fecal and serum samples for metabolomics analysis

The feces of each group of mice in the 10th week were collected by massaging the abdomen method and stored in an ultra-low temperature refrigerator at −80 °C. Weigh 50 mg of the collected feces in a 2 mL centrifuge tube, add 500 μL of phosphate buffer containing 0.05% TSP-labeled heavy water (10% heavy water, pH = 7.4), vortex mixing for 15 s, freeze and thaw repeatedly three times with liquid nitrogen, homogenize the samples for 2 min with an S10 handheld high-speed homogenizer and centrifuge the samples for 15 min at 4 °C and 12,000 r/min, to obtain the feces supernatant sample. Repeat the above procedure for the remaining sediment, combine the supernatants obtained by centrifugation, and then centrifuge the samples at 4 °C for 20 min at 16,000 r/min to obtain the fecal supernatant samples of each group of mice in the 10th week. A total of 550 μL of the supernatant samples obtained were added into a 5 mm NMR tube, and the samples were shaken well to be measured.

(2) Parameters used for ^1^H-NMR analysis of sample

The prepared fecal samples of the above mice were placed on a Bruker AVANCE 500 MHz NMR spectrometer (Bruker, Billerica, MA, USA) with Prodigy liquid nitrogen cryoprobe for NMR mapping. The water peaks were pressed by pre-saturation, and the NOESY pulse sequence was used. The specific parameters were set as follows: the number of scans NS = 64, the temperature was 25 °C, the spectral width SWH = 10,000 Hz, and the measurement frequency SF = 500.13 MHz, chirp delay D1 = 2.0 s, sampling points TD = 65,536, sampling time AQ = 3.277 s, fixed echo time D20 = 2 ms, and cycle number L4 = 16.

(3) Data processing and multivariate statistical analysis of sample NMR tests

Fecal sample data were imported into Mestre Nova 14.2 (Mestrelab Research SL, Santiago de Compostela, Spain) after all tests were completed, multiplied by an exponential window function with a broadening factor of 0.3 Hz before Fourier transformation, and then manually adjusted to phase at baseline. The fecal spectra were calibrated with the methyl resonance at TSP (δ0.0) as a reference, and to avoid interference from residual water, the integration of the water peaks at δ4.70–5.15 ppm in the fecal spectra was removed, and the region of the chemical shift interval δ0.00–9.00 ppm was integrated into segments at δ0.002. The results of the integration were normalized.

Multivariate statistical analysis of the sample assay data was performed using Metaboanalyst 5.0 (https://www.metaboanalyst.ca/, accessed on 1 January 2025). Principal Component Analysis (PCA) was used to observe the overall distribution of the sample data, and Orthogonal partial least squares discriminant analysis (OPLS-DA) was used to identify the differences between groups of sample data to ensure the stability of the model. Differential metabolites were screened according to the variable projected importance (VIP > 1) and in combination with independent-samples *t*-test *p* < 0.05. Differential metabolites with a correlation coefficient (|r|) >0.4 and *p* < 0.05 were considered metabolites relevant to protein intake when present. Then, the differential metabolites with correlation were selected to implement the metabolic pathway analysis, and the impact threshold of the metabolic pathway was VALUE > 0.08.

### 2.6. Comprehensive Quantitative Scoring Method Construction and Comprehensive Evaluation of Test Samples

To comprehensively characterize the effect of dietary intervention on the anti-aging of each group of experimental animals, based on the theory of multivariate statistical analysis, we established a comprehensive quantitative scoring method. PCA was used to analyze the various mouse aging indicators quantitatively, and the data were extracted from the three dimensions of behavioral, oxidative stress, and inflammatory factors and processed through dimensionality reduction, standardization, and dimensionless processing to obtain the comprehensive quantitative evaluation result *F_sum_* [27].

(1) $$F_{sum}=\frac{V_{1}}{M}\left(\sum_{k=1}^{4}a_{i}X_{i}\right)+\frac{V_{2}}{M}\left(\sum_{k=1}^{4}b_{i}X_{i}\right)+\frac{V_{3}}{M}\left(\sum_{k=1}^{4}c_{i}X_{i}\right)+\frac{V_{4}}{M}\left(\sum_{k=1}^{4}d_{i}X_{i}\right)+\frac{V_{5}}{M}\left(\sum_{k=1}^{5}e_{i}X_{i}\right)$$

where *V_i_* denotes the variance explained ratio of the sample under each principal component; *M* denotes the cumulative variance of the sample; *a_i_*, *b_i_*, *c_i_*, and *d_i_* denote the first, second, third, and fourth components, respectively; and *X_i_* denotes the cumulative variance of the sample.

### 2.7. Statistical Analysis

SPSS26.0 (SPSS Inc., Chicago, IL, USA) software was used to statistically analyze the experimental data, which are expressed as the mean ± standard deviation. The normality of the data was assessed using the Shapiro–Wilk test. Depending on the distribution of the data, parametric or non-parametric statistical tests were applied accordingly. For normally distributed data, one-way ANOVA followed by Tukey’s multiple comparison test was used to compare differences between groups, selected for its robustness in detecting group-wise differences in biological experiments with multiple interventions. For non-normally distributed data, mainly metabolomics data, the Kruskal–Wallis test followed by Dunn’s multiple comparison test was applied, chosen to accommodate non-parametric distributions common in small-sample biological datasets. *p* <  0.05 was considered statistically significant.

## 3. Results

### 3.1. Effects of the Intervention of Dietary Protein Content Changes on the Behavioral Characteristics of Mice

**Figure 2:**
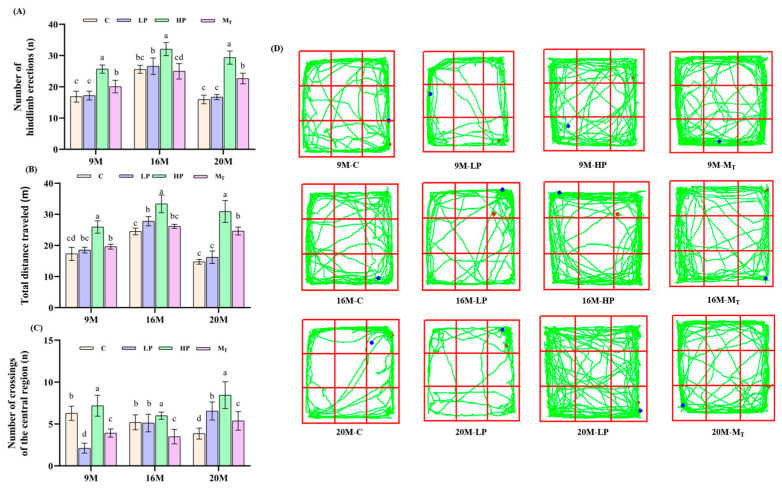
Effects of diets with different protein levels on the exploratory ability of mice: (**A**) total distance traveled; (**B**) number of hindlimb uprights; (**C**) number of central region entries. (**D**) Movement trajectories of different groups of mice. The red color in the figure represents the starting point, and the blue color represents the end point. Different letters indicate statistically significant differences within groups at that age (*p* < 0.05).

The results of the mouse sample absenteeism experiment are shown in Figure 2.

As shown in Figure 2A, the number of erections of the hind limbs in the 9-month-old high-protein group (9 M-HP) mice amounted to 26 ± 2.3, which was a significant increase of 52.9% (*p* < 0.01) compared with 17 ± 1.5 in the 9 M-C control group. That in the 16 M-HP group amounted to 32 ± 2.8, which was an enhancement of 28% (*p* < 0.01) compared with 25 ± 2.1 in the 16 M-C control group. That in the 20 M-HP group was 30 ± 2.8, a 100% improvement (*p* < 0.01) over the 20 M-C control group of 15 ± 1.2, which was the most significant improvement. It recovered to 23 ± 2.1 in the 20 M-M_T_ group, which was 53.3% (*p* < 0.01) elevated compared with the 20 M-C group but significantly lower than 30 ± 2.8 in the 20 M-HP group (*p* < 0.01). Notably, the LP group did not show significant improvement at all ages. The above results suggest that the 10-week high-protein intervention significantly reduced the anxiety level in mice. By contrast, the later 5-week HP intervention was still able to partially reverse LP-induced anxiety in the older age stage.

As shown in Figure 2B, the total distance traveled by the 9-month-old high-protein (9 M-HP)-fed mice was 25.94 ± 1.3 m, and that of the 9 M-C control group was 17.31 ± 1.1 m, with an increase of 48.7% in the former group compared with the latter (*p* < 0.01). That of the 16 M-HP group was the furthest, 33.37 ± 1.5 m, which was 36.15% higher than that of the 16 M-C control group of 24.51 ± 1.2 m (*p* < 0.05), while the 20 M-HP group moved the furthest total distance of 33.93 ± 1.6 m, which was 129.88% (*p* < 0.01) higher than that of the 20 M-C control group of 14.76 ± 0.34 m. The 9 M-M_T_ and 16 M-M_T_ groups moved the furthest distance of 19.63 ± 0.56 and 26.15 ± 0.48 m, respectively, which were slightly but not significantly higher than the control level (*p* > 0.05), while the 20 M-M_T_ group moved a distance of 24.62 ± 0.76 m, which was a 66.8% enhancement compared with the 20 M-C group of 14.76 ± 0.34 m (*p* < 0.01), but also significantly lower compared with the 20 M-HP group. The above results indicated that the 10-week high-protein intervention significantly enhanced locomotor performance, whereas the later 5-week HP intervention also partially reversed the LP-induced locomotor deficits in the old age stage, but the effect gradually decayed with age.

As shown in Figure 2C, the number of times the mice explored the central region in the 20 M-HP group amounted to 8 ± 1.3, which was a significant enhancement of 100% compared with 4 ± 0.8 in the control group (*p* < 0.01) and corresponded to the degree of enhancement of 129.88% of the total distance traveled, suggesting that the high-protein diet significantly mitigated the decline in exploratory ability in the aged mice. The number of times the mice explored the central region was 7 ± 1.1 for the 20 M-LP group and 7 ± 1.1 for the 9 M-LP group. That for the 9 M-M_T_ group was 4 ± 0.7, which was a 33.3% decrease compared to the control 9 M-C group of 6 ± 0.9 and did not reach a significant difference (*p* > 0.05), suggesting that the dynamic adjustment strategy had no improvement effect on the central exploratory behavior in young mice. That of the 16 M-M_T_ group was 4 ± 0.8 compared to the control 16 M-C group of 5 ± 0.8, which was a non-significant decrease (*p* > 0.05), suggesting that the dynamic intervention is not practical in modulating exploratory behavior in the middle-age stage. By contrast, that of the 20 M-M_T_ group of 6 ± 1.0 improved by 50% (*p* < 0.01) compared with the control group, which had some effect but was significantly lower than the improvement effect of 8 ± 1.3 of the 20 M-HP group.

From the mouse movement trajectories (Figure 2D), the percentage of movement trajectories covering the central region in the HP group of all ages was as follows: 9 M, 30%; 16 M, 35%; and 20 M, 40%. Compared with the control group of 20 M-C, the number of times the mice entered the central region in the 20 M-HP group increased by 100%, reflecting the reduction in anxiety level in each group after the intervention (*p* < 0.01), whereas the trajectories of the LP group were especially concentrated in the marginal region. The movement trajectories of the 9 M-LP and 20 M-LP groups covered more than 70% of the edge region, with short and repetitive paths, suggesting that the low-protein diet elevated the anxiety level of the mouse samples. By contrast, the movement trajectory of the 20 M-M_T_ group was shifted toward the central region. The total distance traveled was elevated by 66.8% compared with that of the 20 M-LP group (*p* < 0.01). However, it was still lower than that of the 20 M-HP group (*p* < 0.05), which indicated that the later 5-week HP intervention could partially restore the exploratory behavior but could not completely reverse it.

The above results indicated that female mice of all ages improved all three ability parameters after 10 weeks of HP dietary intervention. By contrast, the 5-week LP intervention showed adverse changes in the middle- and old-aged groups. The later 5-week HP intervention group partially reversed the decline in behavioral ability caused by pre-LP. Still, the effect was age-differentiated, and the medium-protein group generally did not perform as well as the high-protein group. The results were significantly different from those of our team’s study using male rats [17], especially the 20 M group, which showed the most remarkable difference in performance, which predicts that female mice are less sensitive to the metabolic changes induced by fluctuations in protein nutrient levels than are males.

### 3.2. Effects of Dietary Protein Content Change Intervention on Learning Memory Ability of Mouse Samples

**Figure 3:**
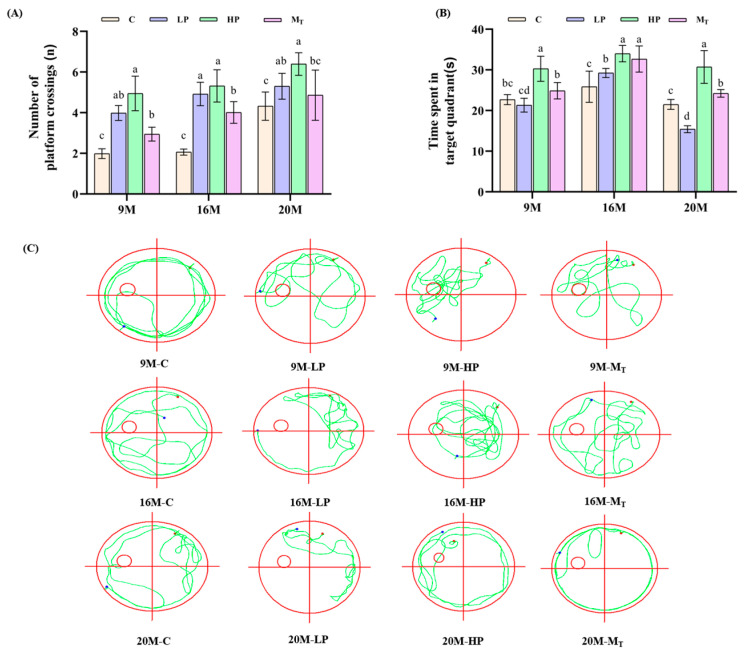
Effects of diets with different protein contents on spatial exploration ability of mice: (**A**) Number of crossing platforms. (**B**) Time spent in the target quadrant. (**C**) Movement trajectories of different groups of mice. The red color in the figure represents the starting point, and the blue color represents the end point. Different letters indicate statistically significant differences within groups at that age (*p* < 0.05).

The Morris water maze test results for each group of mice are shown in Figure 3.

As shown in Figure 3A, the 20 M-HP group crossed the platform the most times, 6.39 ± 0.46, which was a significant increase of 47.15% compared with the 20 M-C group of 4.32 ± 0.56 (*p* < 0.05); the 16 M-HP group crossed the platform the most times, 5.31 ± 0.78, which was a highly significant increase compared with the 16 M-C group of 2.06 ± 0.34 (*p* < 0.05), 157.77% (*p* < 0.01); and the 9 M-HP group traversed the platform 4.95 ± 0.89 times with a highly significant improvement of 150% (*p* < 0.01) over that of 9 M-C of 1.98 ± 0.14. This indicated that the HP group of all ages could significantly (*p* < 0.01) improve the spatial learning memory ability, and the effect did not decline but enhanced with age.

In terms of the mice’s residence time in the target quadrant (Figure 3B), there was no difference in the escape latency of the mice before training, and most of them found or were unable to find the platform around the 60 s mark. After learning, the latency of 38.3 ± 3.9 s in the 20 M-HP group was shortened by 35.2% (*p* < 0.01) compared with 59.1 ± 4.3 s before learning, and the latency of 40.5% (*p* < 0.01) and 47.3% (*p* < 0.01) in the 16 M-HP and 9 M-HP groups, respectively, compared with that before learning, indicated that the high-protein diet could delay the decline in exploration ability, and the younger the age, the better the delaying effect. In the 20 M-LP group, the final test latency of 58.9 ± 1.5 s showed the least change of 1.67% compared to the initial 59.9 ± 1.2 s, which indicated that the low-protein diet had limited or unfavorable effects on the improvement in spatial learning ability in the aged mice.

From the trajectory of the mice (Figure 3C), the HP group had a clear advantage, with the swimming path pointing straight to the platform after entering the water. The escape latency of the young 9 M-HP group of 28.7 ± 3.2 s was 47.1% shorter than that on the first day (*p* < 0.05) (Supplementary Table S1). The latency of the older 20 M-HP group of 38.3 ± 3.9 s was 35.2% shorter than that on the first day for this group (*p* < 0.01). By contrast, the overall path of the LP group was blindly meandering, and the latency period did not change significantly. For the M_T_ group, the path efficiency improved compared with the LP group, but the target quadrant residence time was still lower than that of the HP group, suggesting that the low-cognitive-level state was partially reversed after the later 5-week intervention but could not be restored to that of the younger age group.

### 3.3. The Effect of Dietary Protein Content Change Intervention on Neurons in the Hippocampal Region of Mice

**Figure 4:**
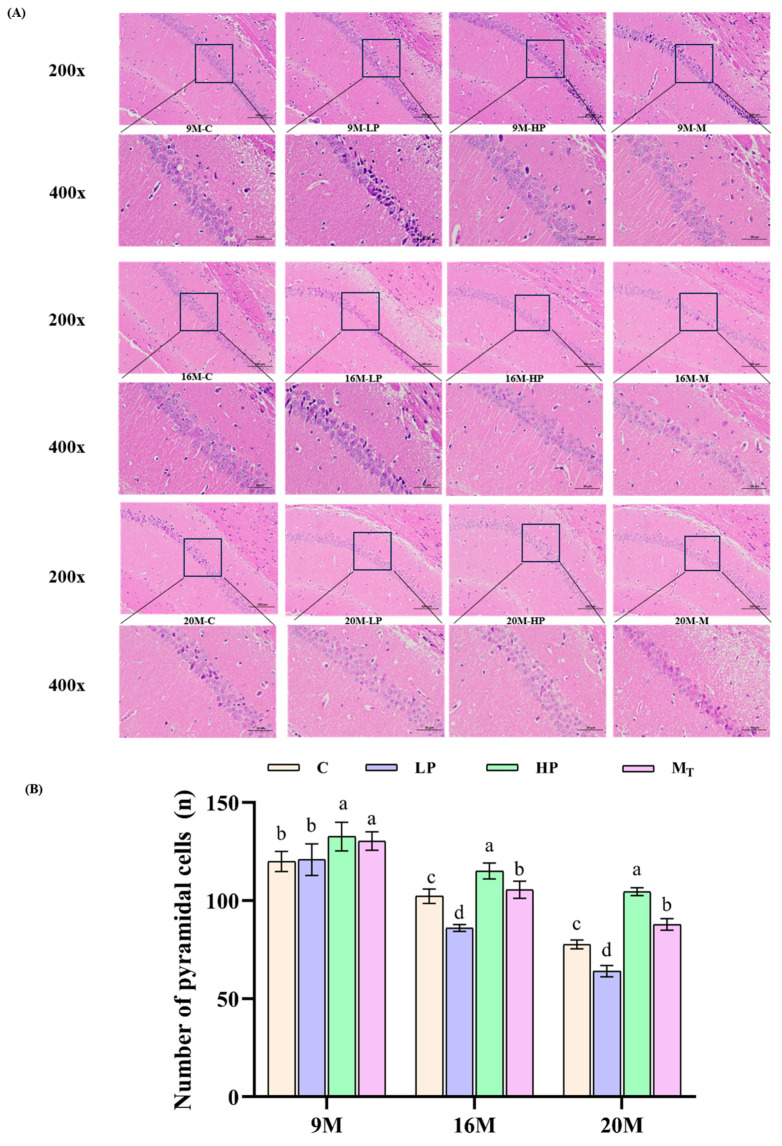
Therapeutic effects of diets with different protein content on inflammation in the mouse brain. (**A**) Microscopic images of the CA1 region of the hippocampus stained with different magnifications of H&E; (**B**) quantitative analysis of pyramidal cells in specific regions of the CA1 region of the hippocampus. Different letters indicate statistically significant differences within the group at that age (*p* < 0.05).

The results of tissue section observation of the mouse samples are shown in Figure 4, which shows that the HP group had the highest number of neuronal pyramidal cells per unit area of hippocampal CA1 region among the samples of all ages, in which the density of pyramidal cells (in the rectangular box) of the 20 M-HP group at a magnification of 400 reached 104.6 ± 5.8 per mm^2^, which was 32.9% higher than that of the 20 M-C group (*p* < 0.01). This suggests that the HP group of all ages exhibited better mitigation of neuronal loss in the CA1 region of the hippocampus associated with aging by high-protein nutrition. It was also observed that the 16 M-LP group showed a 15.83% decrease in neurons compared to the 16 M-C group. By contrast, the 20 M-LP group had 64.08 ± 8.2 neurons/mm^2^, which was a 17.51% decrease in neurons compared to the 20 M-C group (*p* < 0.05), which indicated that neuronal decline deepened in the LP group with age, suggesting that low-protein diets bring about more pronounced neurological damage in the aging organism. There was no significant difference (*p* > 0.05) in the 9 M-M_T_ group with 128.90 ± 5.1 neurons/mm^2^ compared to the 9 M-C group with 123.45 ± 4.5 neurons/mm^2^, while the 16 M-M_T_ group (105.40 ± 3.90 neurons/mm^2^) showed a difference of 10.32% *(p* > 0.05) compared to the control group, indicating that the female mouse organism’s ability to repair neurological injuries caused by low protein decreases markedly with the increase in the degree of aging. This predicts that females after the age of 60 years should be more cautious in avoiding protein nutritional deficiencies because females on a low-protein diet have a significantly reduced ability to recover from the damage to the neurological system caused by these diets, as well as from the resulting memory impairment.

### 3.4. Effects of Dietary Protein Content Change Intervention on Serum Inflammatory Markers and Brain Antioxidant Capacity in Mice

**Figure 5:**
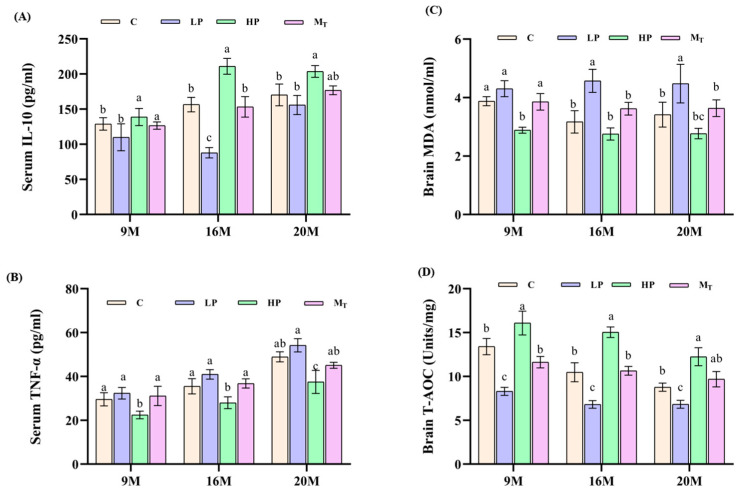
Effects of diets with different protein contents on oxidative stress and inflammation in mouse brain. (**A**,**B**) Effects of varying protein content diets on IL-10 and TNF-α in serum of senescent mice. (**C**,**D**) Effects of varying protein content diets on MDA and T-AOC in mouse brain tissue. Different letters indicate statistically significant differences within groups at that age (*p* < 0.05).

From the results of the detection of verified markers in serum samples of mice in each group (Figure 5A,B), the level of IL-10 (positive indicator) of mice in the 20 M-HP group was 200.5 ± 15.2 pg/mL higher than that of the 20 M-C group by 14.5% (*p* < 0.05). By comparison, the level of TNF-α (downbeat indicator) of this group was 38.7 ± 2.8 pg/mL lower than that of the 20 M-C group by 19.7% (*p* < 0.01), and metabolomics analysis showed that the fecal-positive indicator butyric acid content was elevated by 102% in the 20 M-HP group compared with the 20 M-C group (*p* < 0.01), which predicted that the HP diet mitigated inflammatory aging [28]. The TNF-α level was 52.5 ± 3.5 pg/mL in the 20 M-LP group, which was elevated by 8.9% compared with that of the 20 M-C group (*p* < 0.05), suggesting an increase in inflammation in the elderly LP group [29]. From Figure 5C,D, the brain MDA (reverse indicator) level of mice in the 20 M-HP group was 2.77 ± 0.31 nmol/mg, which was 19.01% lower than that of the 20 M-C group (*p* < 0.05). By comparison, the brain T-AOC (positive indicator) content of the mice in the same group increased by 39.68% compared with that of the control group (12.25 ± 1.3 in the 20 M-HP group vs. 8.77 ± 0.6 in the 20 M-C group). The MDA level of mice in the 20 M-LP group was 4.47 ± 0.7 compared to the 20 M-C group (*p* < 0.05), while the T-AOC activity of mice in the 20 M-HP group was 6.8 ± 0.5 vs. 8.8 ± 0.7 in the 20 M-C group (*p* < 0.05), which indicated that the antioxidant capacity of mice in the LP group decreased or was imbalance. The T-AOC content of 9 M-M_T_ and 9 M-C, 16 M-M_T_, and 16 M-C groups recovered to 91.7 and 91.96% before and after the intervention, respectively. By contrast, the corresponding T-AOC content of the 20 M-M_T_ and 20 M-C groups recovered to 104.5%, which suggests that the late intervention is sensitive to the female mouse samples in terms of total antioxidant capacity indexes and is not limited by age. On the contrary, the older the age, the better the effect. In other words, the HP diet for 5 weeks also significantly improved the recovery of total antioxidant capacity, showing a dose-time-dependent effect (Figure 5C,D).

### 3.5. Effect of Dietary Protein Content Change Intervention on Fecal Metabolites in Mice

**Figure 6:**
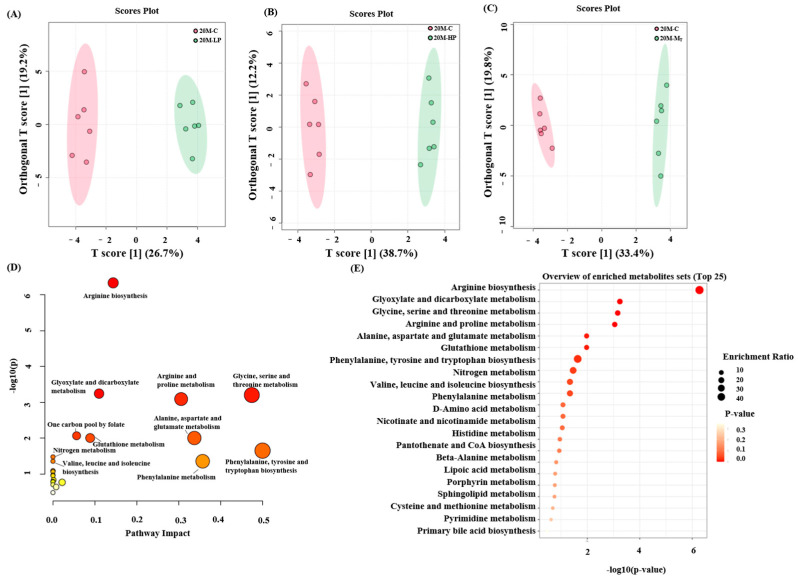
Fecal metabolite NMR assay results and their and metabolic pathway analysis in aged 20 M group mice. (**A**–**C**) OPLS-DA analysis of 20 M group; (**D**) KEGG pathway enrichment scatter plot of 20 M group; (**E**) metabolic pathway analysis of 20 M group.

In this study, mouse fecal metabolites were examined using NMR technology, and their metabolic pathways were analyzed based on the results (Figure 6E, Supplementary Figures S1E and S2E). Thirty-three metabolites were detected in mouse fecal samples. Orthogonal partial least squares discriminant analysis (OPLS-DA) was used to compare the metabolite differences between groups. The results showed significant differences in fecal metabolites between the HP, LP, M_T,_ and C groups of mice of all ages. The R^2^Y and Q^2^ of the data of each group were above 0.9 and 0.8 (by 100 permutation test), respectively, which indicated that each group had valid predictive power and significant explanatory power [30].

Next, enrichment and pathway analyses of the 30 metabolite analyses of the 20 M group were performed, and the results (Figure 6A–C) revealed that most of the metabolites were focused on valine, leucine, and isoleucine biosynthesis; valine, leucine, and isoleucine degradation; β-alanine metabolism; glycine, serine, and threonine metabolism; and phenylalanine, tyrosine, and tryptophan biosynthesis, among other metabolites.

**Figure 7:**
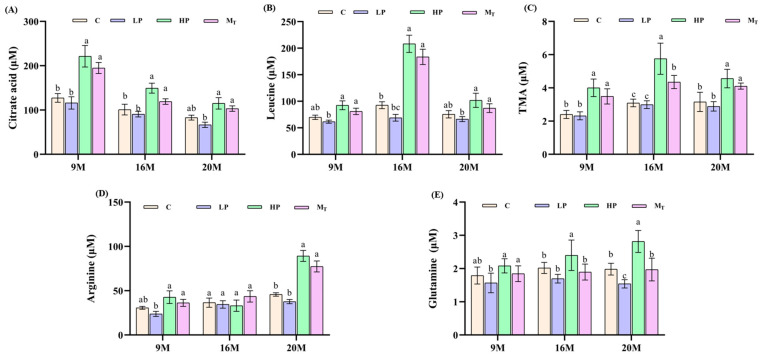
Five specific metabolite contents. (**A**) Citric acid content; (**B**) leucine content; (**C**) TMA content; (**D**) arginine content; (**E**) glutamine content. Different letters indicate statistically significant differences between groups (*p* < 0.05).

Accordingly, the present study was conducted to investigate the changes in the levels of citric acid, leucine, trimethylamine, arginine, and glutamine in the fecal samples of mice with different treatments. Among the samples of mice in the 9 M group, the citric acid content was higher in the samples of HP and M_T_ groups (Figure 7A), and the citric acid content of samples in the 9 M-HP group amounted to 219.3 ± 15.6 μM, followed by 200.8 ± 14.5 μM in the 9 M-M_T_ group and only 120.5 ± 10.2 μM in the 9 M-C group, which was significantly higher in the 9 M-HP and 9 M-M_T_ groups than that in the 9 M-C group *(p* < 0.05). By contrast, the citric acid content of 115.2 ± 9.8 μM in the samples of the 9 M-LP group was slightly lower than that of the control group (*p* > 0.05). Citric acid is an important intermediate metabolite in the TCA cycle, which is closely related to the inflammatory regulation potential of the body. Fecal citrate levels in the 9 M group may reflect gut microbial carbohydrate metabolism rather than direct host TCA cycle activity, suggesting an indirect association with energy metabolism. By contrast, low-protein interventions do not have this effect. For 16-month-old mice, leucine levels were significantly higher in samples from the HP and M_T_ groups (Figure 7B), with leucine levels of 198.9 ± 12.3 μM in the 16 M-HP leucine group and 180.5 ± 11.5 μM in the 16 M-M_T_ group, both significantly higher than the control (*p* < 0.05) leucine levels of 85.7 ± 7.8 μM in samples from the 16 M-C group (*p* < 0.01). The level of 70.2 ± 6.5 μM in the 16 M-LP group was lower than that of the control group. Leucine is an important component of the branched-chain amino acids (BCAAs). The high content level and enhanced metabolism caused the upregulation of branched-chain enzyme activity to dominate the catabolism of BCAAs, suggesting that the metabolism of high-protein BCAAs was enhanced in the middle-aged group [31], that female mice in the middle-aged stage may require more energy metabolism flexibility due to fluctuating estrogen levels (pre- and post-menopause), and that the degradation of BCAAs coincidentally provides this adaptation [32]. Meanwhile, trimethylamine (TMA) levels (Figure 7C) were as high as 5.9 ± 0.6 μM in the HP group (*p* < 0.05), moderate at 4.5 ± 0.5 μM in the M_T_ group (*p* < 0.05), and basically unchanged at 3.0 ± 0.3 μM in the LP group (*p* > 0.05) versus 3.2 ± 0.4 μM in the control group, whereas the absence of change in the LP group suggests that the low-protein diet did not significantly activate the relevant metabolic pathways [33]. In the 20 M group, arginine levels were significantly higher in samples from the HP and M_T_ groups (Figure 7D), at 89.3 ± 6.8 and 78.5 ± 6.2 μM, respectively, which were significantly higher than those in the 20 M-C group, at 45.8 ± 5.1 μM, (*p* < 0.01), and in the LP group, at 38.2 ± 4.5 μM, which was lower than those in the control group. At the same time, the glutamine level of 2.8 ± 0.3 μM in the HP group was also nearly 1.5-fold higher (*p* < 0.05) compared with 1.9 ± 0.3 μM in the control group (Figure 7E). This suggests that the aged stage (20 M) will respond to fluctuations in protein intake after HP intervention mainly by upregulating the amino acid biosynthetic pathway, indicating that female mice are still able to maintain physiological homeostasis in old age through specific metabolic adjustments, although their metabolic responses may not be as sensitive as those of males.

The analysis of the metabolite characteristics of the mouse samples in this study showed that the fecal citrate levels in the 9 M group may reflect gut microbial carbohydrate metabolism rather than direct host TCA cycle activity, suggesting an indirect association with energy metabolism. At the same time, the energy metabolism of middle-aged and old mice was also enhanced at the amino acid level when they received the high-protein diet. This result may produce “suitable high-protein nutrition” for people. The judgment that it can prevent the decline in protein synthesis caused by aging and thus slow muscle loss in the elderly population provides new evidence. The above results also indicate that dietary intervention with different protein levels can interfere with the body’s energy metabolism by interacting with correlated differential metabolites and affecting the body’s health.

### 3.6. Comprehensive Quantitative Evaluation of the Anti-Aging Effect of Dietary Protein Content Change Intervention on Mice

**Figure 8:**
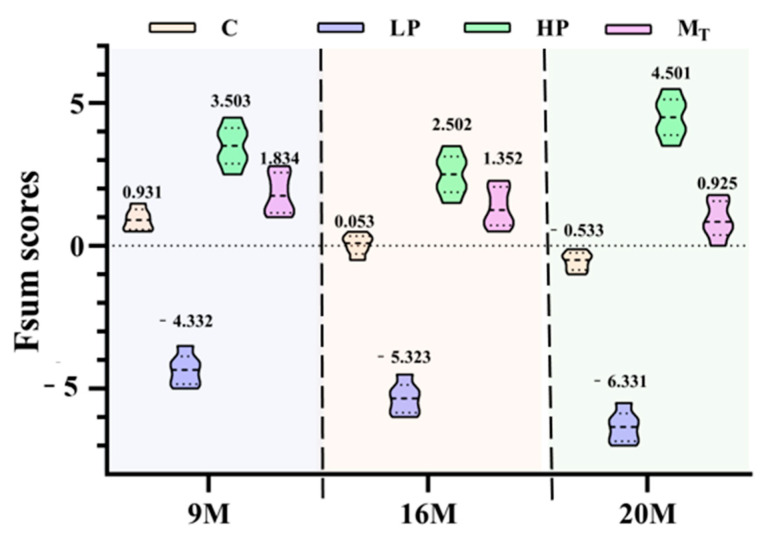
Fsum scores violin plot of anti-aging effect in mice.

To quantitatively characterize the overall effect of the dietary protein content change intervention on the impact of aging in experimental animals, we constructed a set of comprehensive health scoring methods. The specific operation is to integrate the three dimensions of behavioral, oxidative stress, and inflammatory factors (nine indicators), and then standardize the raw data of each indicator to obtain the dimensionless data before PCA statistical analysis. Before the analysis, we used the Kaiser–Meyer–Olkin (KMO) test and Bartlett’s test to determine whether the data met the requirements of PCA. The method of a previous study [16] calculated the results of KMO = 0.757 and Bartlett’s test (*p* < 0.05), which met the criteria of KMO > 0.6 and Bartlett’s test (*p* < 0.05) [27]. Accordingly, PCA was performed to determine a comprehensive quantitative score of the nine indicators, and the five principal components with eigenvalues greater than 0.9 were extracted, at which point the variance accumulation rate reached 75.722%, referring to Pinto’s report [23]. When the variance accumulation rate exceeded 70%, subsequent analysis could be performed. The standardized data and loading factors (Table 1 and Table 2) were plugged into Equation (1). Then, GraphPad Prism 8.0 software (GraphPad Software, San Diego, CA, USA) was further used to visualize the comprehensive quantitative scores of the mice in each group. Fsum score violin plots were obtained for the anti-aging effects of different protein nutritional modalities on mice (Figure 8).

The violin plots can visualize and image the distribution of the total scores of mice in each intervention group and also reflect the detailed distribution of the health status of different individuals within each group after the intervention of various proteins.

As shown in Figure 8, the three composite score violin plots of mice in group C (control group yellow) were in the form of lower storage tanks (one thick at the bottom and two thick at the top). The health composite scores were 0.931 (group 9 M), 0.053 (group 16 M), and −0.533 (group 20 M), with the corresponding upper and lower quartile differences of 0.472, 0.486, and 0.459; the distribution was relatively concentrated. According to the distribution properties of the violin plot, this result indicates that the variability in the health status of the organisms of the three control groups of mice is relatively tiny, but the distribution of the health scores of the mice within the different age groups also varies. The distribution of the two aging mice of 16 and 20 months of age is minimal, but the distribution of the two is different from those of 9 months of age.

The high-protein group (HP, yellow-green) had the highest composite quantitative scores, which predicted the excellent effect of the high-protein intervention in improving the organic health status of female mice, with specific scores of 3.503 (9 M group), 2.502 (16 M group), and 4.501 (20 M group), respectively. The violin plots of the three HP groups showed an overall upper and lower symmetrical violin shape. The upper and lower quartile differences were 0.892 (9 M group), 0.901 (16 M group), and 0.945 (20 M group), respectively, with the distribution of the composite quantitative scores relatively dispersed. The scoring values fluctuated within a wide range, which reflected that there were specific individual differences in the effects of high-protein interventions on the health status of the organisms and also signified that the microorganisms of the mice had a significant fluctuation in health status. The scores of the HP group were significantly higher than those of the other groups, indicating that the high-protein intervention had a better overall effect in improving the health status of female mice.

The mice in the three low-protein groups (LP, light purple) had the lowest overall quantitative scores. All three violin plots were in the shape of low milk drink bottles, with scores of −4.332 (group 9 M), −5.323 (group 16 M), and −6.331 (group 20 M), and upper and lower quartile differences of 0.659 (group 9 M), 0.679 (group 16 M), and 0.671 (group 20 M), respectively, and the distribution of the scores was relatively concentrated. The relatively centralized distribution of score fractions suggests that the low-protein intervention resulted in a generally low health status and poor overall anti-aging effect in the mouse samples. The violin plots of the distribution of the quantitative health status scores of the three groups of mice (Group M_T_, purplish red) were vase-shaped, in which the 9- and 16-month-old groups were similarly shaped, with both having shapes of small upper mouths, thin waists, and thicker lower mouths. By contrast, the 20-month-old group had the shape of a vase that had a narrowed bottom, a slightly bulging belly at the upper level, a wider waist, a thinner neck, and a slightly open mouth. The health scores were 1.834 (9 M group), 1.352 (16 M group), and 0.925 (20 M group), with upper and lower quartile differences of 0.789 (9 M group), 0.799 (16 M group), and 0.802 (20 M group), respectively, reflecting a more consistent response of individuals to the health conditions in the model intervention groups, and a composite evaluation score that was in the range of those of HP and C. The results also reflected that the overall evaluation score was between those of HP and C, which also reflected the successive fluctuation in health status caused by 5 weeks of low protein in the first period and 5 weeks of high protein in the second period, achieving a better anti-aging effect.

### 3.7. Interaction of Fecal Metabolites with Behavioral and Antioxidant Indicators and Inflammatory Markers

**Figure 9:**
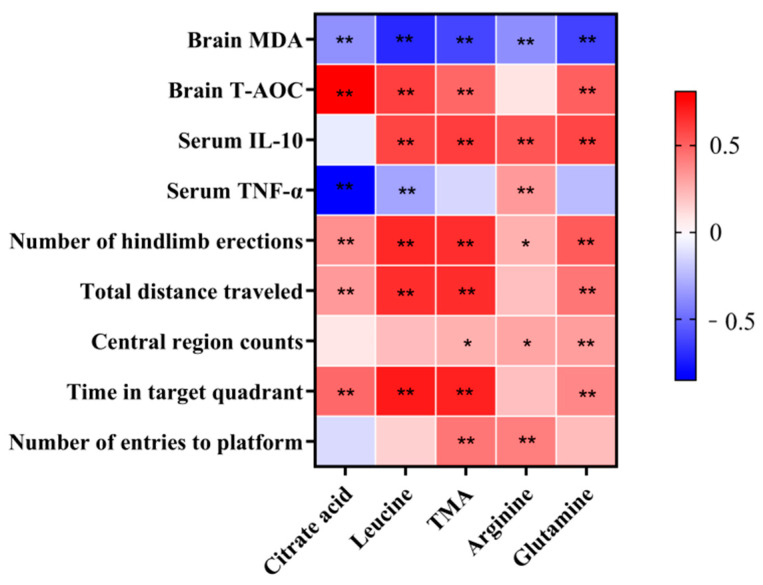
Heat map of correlations between fecal metabolites and aging-related parameters. Asterisks indicated statistically significant differences (* *p* < 0.05, ** *p* < 0.01).

In order to characterize the anti-aging effects of different dietary interventions, correlations between the characteristic fecal metabolites reflected after the intervention and each aging-related parameter were explored based on correlation coefficients (Figure 9). All five characterized fecal metabolites were negatively correlated with brain MDA and positively correlated with the number of hindlimb erections. The brain T-AOC, total distance traveled, and time in target quadrant were positively correlated with the other characterized metabolites except arginine. Serum IL-10 was positively correlated with all metabolites except citrate acid; serum TNF-α was positively correlated with arginine and negatively correlated with citrate acid and leucine. In addition, the number of entries into the central region was positively correlated with TMA, arginine, and glutamine, and the number of entries to platform was positively correlated with TMA and arginine.

## 4. Discussion

In the present study, a high-protein (HP) diet was imposed on C57BL/6J female mice in the young (9 M), middle-aged (16 M), and old (20 M) stages and was found to exhibit a significant age-dependent anti-aging effect, which was especially prominent in the old group (20 M). The 20 M-HP group showed an improvement in the distance traveled before and after the intervention of 129.9% (*p* < 0.01), with a gain far exceeding that of the young 9 M-HP group (48.7%, *p* < 0.05), and also significantly higher than 75.3% in the male model [17], suggesting that the potential for recovery of locomotor ability is more substantial in female mice in the old age stage. Although female mice may exhibit less pronounced metabolic pathway adjustments compared to males (e.g., a preference for arginine biosynthesis over BCAA degradation), the HP diet still significantly enhances motor and cognitive performance in aged females, as evidenced by a 129.9% improvement in travel distance (*p* < 0.01) in the 20 M-HP group. This suggests that functional improvements can occur independently of the sensitivity of metabolic re-adaptation. Elevated fecal leucine levels in the 16 M-HP group (198.9 ± 12.3 μM, *p* < 0.01) likely result from enhanced microbial BCAA metabolism in response to the HP diet rather than host biosynthesis, given that BCAAs are essential amino acids derived from diet or protein turnover. Fecal amino acid profiles, such as increased BCAAs in the 16 M-HP group, reflect dietary protein metabolism and gut microbial activity, not host biosynthesis. These findings in mice suggest potential dietary implications for humans but may not be directly extrapolated to human metabolic pathways.

Paddon et al. [34] indicated that in the old age stage, an adequate supply of protein is crucial for maintaining muscle mass and neurological function [35]. There are now several studies recommending that the daily protein intake should reach 1.2–1.5 g/kg of body weight to fully activate metabolic compensation mechanisms and reduce the risk of aging-related dysfunctions such as sarcopenia and cognitive decline [36,37]. The results of the present experiments provide new theoretical support for these ideas.

The results of the Morris experiment in mice obtained in this study showed that HP intervention was effective in improving spatial memory in aged female mice: the escape latency in the 20 M-HP group was shortened by 35.2% compared with the first day of the same group (*p* < 0.01), whereas the results of Zhou et al. [27] tested in the corresponding male mice were only shortened by 22.1%. By contrast, the male model used by Zou et al. [38] showed that the corresponding mice’s latency period was prolonged by 18.5%, suggesting that high-protein nutrition is more effective in aged females than in middle-aged and aged males. In this study, we also found that the brain MDA level of mice in the 20 M-HP group was reduced by 19.0% (*p* < 0.05), and T-AOC activity was elevated by 39.7% (*p* < 0.05) compared to the 20 M-C group, which was superior to Bellant et al. [18] report of a 12% reduction in MDA and a 25.3% elevation in T-AOC in males after intervention with animal proteins (*p* < 0.05).

HP intervention was also able to significantly alleviate neuronal damage in the CA1 region of the hippocampus in female mice: the density of mouse hippocampal pyramidal cells per unit area was elevated by 32.9% in the 20 M-HP group before and after the intervention compared to the control group (*p* < 0.01), which was significantly higher compared to the result of 21.5% (*p* < 0.05) in the male model of Zheng et al. [17], which may benefit from the protection of mitochondrial function mediated by estrogen [33]. The neuronal density of mice in the dynamic mode group (M_T_ group) still recovered to 89.10 ± 3.5 neurons/mm^2^ (11.24% increase compared to the control group, *p* < 0.05) after the intervention in the old age stage. By contrast, it only recovered to 64.50 ± 2.8 neurons/mm^2^ in the male model group [17]. This result again suggests that the elasticity window during which the metabolism of female mice has the potential to recover can be extended up to 20 M, which paralleled Lehallier et al.’s result [22], who suggested that 78 years of age in humans is a metabolic turning point. These differences indicate that the metabolic and neuroprotective effects of HP interventions are stronger in female mice in old age. The reason for this may be related to the activation of metabolic compensation by females through a certain period of high protein intake. By contrast, males are unable to fully utilize the effects of the HP diet due to testosterone-driven differences in proteolysis [32]. The present study further validated the phenomenon that low-protein diets may exacerbate neural damage and reduce repair capacity in a female model of aging, in contrast to previous findings in a male sample [39], suggesting that females, especially older females, are less tolerant to low protein (15.2% longer evasion latency in the 20 M-LP group, *p* < 0.05). The neuronal protective effect is strongly correlated with the level of branched-chain amino acids (BCAAs) [40], which is corroborated by the high abundance of leucine found by metabolomics analysis in the present study on the other side, the genesis of which may be related to the activation of synaptic protein synthesis by the mTORC1-S6K1 pathway [41]. The results of the present study on the 20 M-M_T_ group showed that LP + HP intervention partially reversed the neurological damage caused by pre-LP. Although there is an age limitation to the effect, it still supports the notion that the potential for metabolic restoration resides longer in female mice than in males, which provides a new rationale for the strategy that should be adopted with a gender-stratified intervention.

This study used a comprehensive quantitative scoring method to quantify and characterize the anti-aging effect of dietary protein content changes on natural aging female mice. Unlike previous reports [16,24], we integrated nine aspects of three dimensions and extracted five principal components. The results of this study did not reproduce the better anti-aging effect of low protein in the early stage and high protein in the later stage in male middle-aged and old rats [17], which was also consistent with Wallerer et al. [7], who proposed that a long-term low-protein diet can improve the metabolic health of male mice, which is contradictory, again highlighting the gender difference and providing new evidence for the age-specific advantages of a high-protein diet in female mice. The violin chart shows the distribution of the total score of the mice in each intervention group very intuitively, reflecting the distribution of the total score of the body health status of different individuals in each group after different protein nutrition interventions. Green et al. [2] adopted a similar approach to suggest that the body is undergoing adjustments in energy allocation and metabolic readjustment in middle-aged and young mice. Still, the static high and low protein levels of only male and mixed-sex mice were compared in the study, and age stages were not subdivided. In this study, young (9 M), middle-aged (16 M), and elderly (20 M) mice were divided into three stages, and it was found that the HP intervention effect of the elderly group was strongest, revealing the age-dependent metabolic response of female mice. This focus on female mice models filled the gap and highlighted the complexity of mice’s sex-specific responses again.

In summary, based on the hypothesis that “low-protein intervention before 65 years of age (25.9 months of age) in male SD rats and the conversion to high-protein diet at a later stage can significantly improve sample health scores” [17], and combined with the latest findings of this study on female mice, the team made the following judgments: Equivalent to 34-, 65-, 78-year-old human women, protein content is relatively high nutrition (protein content of 25.49/100 g) supply and can obtain a better anti-aging effect. There does not seem to be a need for anti-aging protein nutrition in men in the following way—a moderate change in protein diet from low to high before and after the age of 65 is conducive to achieving anti-aging effects.

This study has the following limitations: (1) The non-targeted ^1^H-NMR method was used for metabolomic analysis. To gain a more comprehensive understanding of the overall metabolic process of different protein diets, new omics analysis methods such as proteomics and lipid metabolomics can be applied in future studies to explore the anti-aging mechanism of different protein diets from various aspects. (2) In the future, this study can be combined with gut microbiota–host interaction analysis to reveal the gut microbiota-specific effects of protein intervention from more perspectives. (3) This study was conducted on female C57BL/6J mice, and male mice and other strains of mice (such as BALB/c) can be included in the future to compare the metabolic and anti-aging responses between the sexes systematically. Mice intervention was carried out for a more extended period. These findings in mice suggest potential dietary implications for humans but may not be directly extrapolated to human metabolic pathways. In the follow-up experiment, a wider range of ages can be selected to recruit middle-aged and elderly volunteers for dietary intervention, to increase the group size, and to reduce the intensity of protein level changes to further explore the effect of this protein dietary pattern based on nutrition strategies of different ages on the health status of middle-aged and older adults. (4) It is important to acknowledge that aging is a multifactorial process influenced by many variables other than protein intake and to recognize the complexity of the aging mechanism, which involves the interaction of genetics, diet, environment, and lifestyle. This study mainly focused on the effect of protein intake, and in the future, specific protein amino acid composition, digestibility effects, exercise, hormone replacement, and other variables can be included to analyze anti-aging strategies more comprehensively.

## 5. Conclusions

In this study, we conducted behavioral tests, oxidative inflammation tests, metabolomics analysis, and comprehensive quantitative scores by naturally aging female mice at three ages and confirmed that the HP group (25.49/100 g) obtained higher comprehensive quantitative scores and outperformed the M_T_, LP, and C groups at all three ages. In the older 20 M stage especially, the 20 M-HP Fsum scores were highest at 4.501 and significantly improved motor performance. In different diet interventions, the movement distance of the 20 M-HP group was 129.9% higher than that of the 20 M-C group (*p* < 0.01), and cognitive function escape latency was increased by 35.2% (*p* < 0.05). Moreover, neuron density in the hippocampal CA1 region was increased by 32.9% (*p* < 0.05) in aged female mice (20 M). Metabolomics further revealed that older female mice responded to protein fluctuations by upregulating amino acid biosynthesis (arginine was increased by 95.0%, (*p* < 0.05)) rather than BCAA degradation in males, suggesting the need for gender stratification and strains of mice. Female mice had sustained neuro-motor benefits from HP diets at different stages of aging, and their protein requirement dynamics were significantly different from those of males. This study revealed the key role of gender factors in protein nutrition strategies and provided an experimental basis for precision protein supplementation in elderly women.

## Figures and Tables

**Table 1:** Eigenvalues and cumulative variance contribution of health evaluation indexes in mice.

Principal Components	1	2	3	4	5
Eigenvalues	2.214	1.624	1.111	1.054	0.902
Explanatory rate of variance%	23.603	18.044	12.340	11.709	10.028
Cumulative variance contribution%	23.603	41.646	53.986	65.694	75.722

**Table 2:** Factor loading matrix.

No.	Name	PC 1	PC 2	PC 3	PC 4	PC 5
1	Total distance traveled	0.047	0.881	−0.078	−0.177	0.041
2	Number of times hind limb upright	−0.024	0.758	0.286	0.239	0.116
3	Central region counts	0.024	0.111	0.048	−0.022	0.971
4	Time in target quadrant	−0.038	0.181	0.887	−0.021	0.001
5	Number of entries to platform	−0.063	−0.004	0.024	0.903	−0.024
6	Brain T-AOC	0.736	−0.084	0.291	−0.232	−0.257
7	Brain MDA	0.557	−0.130	0.627	0.088	0.115
8	Serum IL-10	0.703	0.039	−0.149	0.336	0.084
9	Serum TNF-α	0.708	0.074	0.025	−0.157	0.070

## Data Availability

The data in this study are available on request from the author. The data is not publicly available due to being a part of an ongoing study.

## Supplementary Materials

The following supporting information can be downloaded at https://www.mdpi.com/article/10.3390/nu17091542/s1, Figure S1: Results of fecal metabolite NMR assay and its metabolic pathway analysis in group 9 M mice. (A–C) OPLS-DA analysis of group 9 M; (D) KEGG pathway enrichment scatter plot of group 9 M; (E) metabolic pathway analysis of group 9 M; Figure S2: Results of fecal metabolite NMR assay and its metabolic pathway analysis in group 16 M mice. (A–C) OPLS-DA analysis of group 16 M; (D) KEGG pathway enrichment scatter plot of group 16 M; (E) metabolic pathway analysis of group 16 M; Table S1: Effect of diets with different protein contents on the escape latency of mice in the water maze. * indicates statistically significant differences between Day 1 and Day 5 (*p* < 0.05).

## Abbreviations

The following abbreviations are used in this manuscript:

HP	High-protein dietary pattern
LP	Low-protein dietary pattern
M_T_	Model test group, switching from LP diet to HP diet at week 5
C	Control protein dietary pattern
20 M	20 months
16 M	16 months
9 M	9 months
TCA	Tricarboxylic acid cycle
BCAAs	Branched-chain amino acids
^1^H-NMR	H Nuclear Magnetic Resonance Spectra
TMA	Trimethylamine
PCA	Principal Component Analysis
KMO	Kaiser–Meyer–Olkin test
OPLS-DA	Orthogonal partial least squares discriminant analysis
MDA	Malondialdehyde
T-AOC	Total antioxidant capacity
IL-10	Interleukin 10
TNF-α	Tumor necrosis factor α
VIP	Variable Importance in Projection
KEGG	Kyoto Encyclopedia of Genes and Genomes
mTOR	mammalian target of rapamycin
ANOVA	Analysis of Variance
NOESY	Nuclear overhauser effect spectroscopy
H&E	Hematoxylin–eosin
