# Supplementary material for: Exploring the Effects of Changes in Dietary Protein Content on Naturally Aging Mice Based on Comprehensive Quantitative Scoring and Metabolomic Analysis
Source: Nutrients. 2025 Apr 30;17(9):1542. doi: 10.3390/nu17091542 (PMC12073357; doi:10.3390/nu17091542)
Supplement: Supplementary file 1 [file nutrients-17-01542-s001.zip › nutrients-3591808-supplementary.pdf]

Article

Exploring the Effects of Changes in Dietary Protein Content on Naturally Aging Mice Based on Comprehensive Quantitative Scoring and Metabolomic Analysis

Xiaohua Zheng [1], Fan Zhou [1], Qinren Zhang [1], Wenxuan Zheng [1], Fengcui Shi [1], Ruiding Li [1], Jingwen Lv [1], and Quanyang Li [1,2,*]

[1] College of Light Industry and Food Engineering, Guangxi University, Nanning 530004, China
[2] Guangxi Key Laboratory of Longevity Science and Technology, Nanning 530200, China
* Correspondence: liquanyang@gxu.edu.cn

Table S1. Effect of diets with different protein contents on the escape latency of mice in the water maze. * indicate statistically significant differences between Day1 and Day5 ($p<0.05$).

Group	Day 1 (s)	Day 5 (s)
9M-C	58.2 ± 5.3	32.5 ± 4.1
9M-LP	59.0 ± 2.1	36.8 ± 3.9*
9M-HP	54.5 ± 4.7	28.7 ± 3.2*
9M-MT	57.1 ± 5.1	30.2 ± 3.5
16M-C	59.2 ± 0.8	45.2 ± 5.3
16M-LP	59.9 ± 0.32	52.6 ± 6.1*
16M-HP	58.7 ± 4.9	34.9 ± 4.5*
16M-MT	59.4 ± 5.2	41.8 ± 4.7*
20M-C	58.9 ± 1.2	53.9 ± 5.2
20M-LP	59.9 ± 1.2	58.9 ± 1.5*
20M-HP	59.1 ± 4.3	38.3 ± 3.9*
20M-MT	59.8 ± 5.0	47.5 ± 4.8*

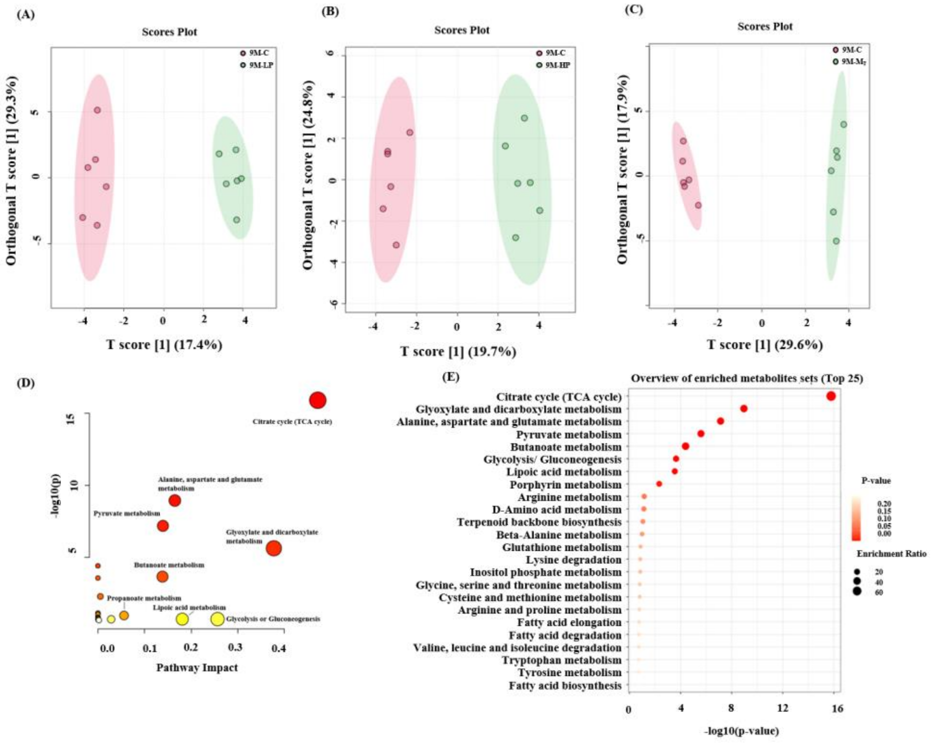

Figure S1. .Results of fecal metabolite NMR assay and its and metabolic pathway analysis in group 9M mice. (A-C) OPLS-DA analysis of group 9M; (D) KEGG pathway enrichment scatter plot of group 9M; (E) metabolic pathway analysis of group 9M

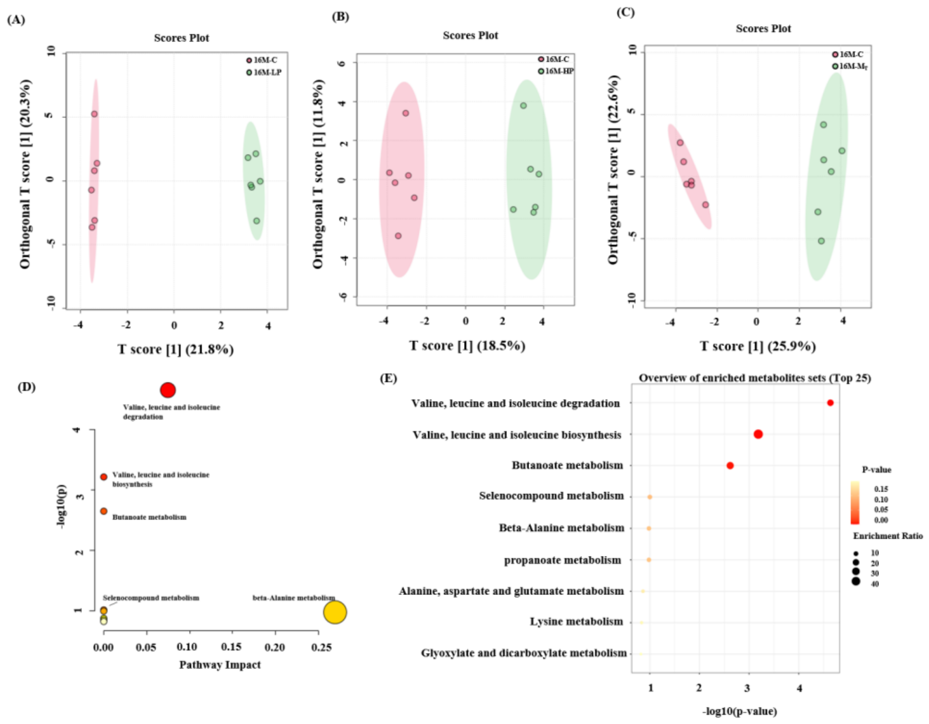

Figure S2. Results of fecal metabolite NMR assay and its and metabolic pathway analysis in group 16M mice. (A-C) OPLS-DA analysis of group 16M; (D) KEGG pathway enrichment scatter plot of group 16M; (E) metabolic pathway analysis of group 16M
